# Mortality associated with administration of high-dose tranexamic acid and aprotinin in primary open-heart procedures: a retrospective analysis

**DOI:** 10.1186/cc9216

**Published:** 2010-08-03

**Authors:** Michael Sander, Claudia D Spies, Viktoria Martiny, Christoph Rosenthal, Klaus-Dieter Wernecke, Christian von Heymann

**Affiliations:** 1 Department of Anaesthesiology and Intensive Care Medicine, Charité-Universitätsmedizin Berlin, Campus Virchow-Klinikum and Campus Charité Mitte, Charitéplatz 1, 10117 Berlin, Germany; 2 SOSTANA (Sophisticated Statistical Analysis) GmbH and Charité-Universitätsmedizin Berlin, Wildensteiner Strasse 27, 10318 Berlin, Germany

## Abstract

**Introduction:**

Antifibrinolytic agents are commonly used during cardiac surgery to minimize bleeding. Because of safety concerns, aprotinin was withdrawn from the market in 2007. Since then, tranexamic acid (TXA) has become the antifibrinolytic treatment of choice in many heart centers. The safety profile of TXA has not been extensively studied. Therefore, the aim of this study was to evaluate safety and efficiency of TXA compared with aprotinin in cardiac surgery.

**Methods:**

Since July 1, 2006, TXA has been administered at a dose of 50 mg/kg tranexamic acid before cardiopulmonary bypass (CPB) and 50 mg/kg into the priming fluid of the CPB. Prior to this, all patients were treated with aprotinin at a dose of 50,000 KIU per kilogram body weight. Safety was evaluated with mortality, biomarkers, and the diagnosis of myocardial infarction, ischemic stroke, convulsive seizures, and acute renal failure in the intensive care unit (ICU), intermediate care unit (IMCU), and hospital stay. Efficiency was evaluated by the need for transfusion of blood products and total postoperative blood loss.

**Results:**

After informed consent, 893 patients were included in our database (557 consecutive patients receiving aprotinin and 336 patients receiving TXA). A subgroup of 320 patients undergoing open-heart procedures (105 receiving TXA and 215 receiving aprotinin) was analyzed separately. In the aprotinin group, a higher rate of late events of ischemic stroke (3.4% versus 0.9%; *P *= 0.02) and neurologic disability (5.8% versus 2.4%; *P *= 0.02) was found. The rate of postoperative convulsive seizures was increased in tendency in patients receiving TXA (2.7% versus 0.9%; *P *= 0.05). The use of TXA was associated with higher cumulative drainage losses (*P*_ANOVA _< 0.01; *P*_time _< 0.01) and a higher rate of repeated thoracotomy for bleeding (6.9% versus 2.4%; *P *< 0.01). In the subgroup of patients with open-chamber procedures, mortality was higher in the TXA group (16.2% TXA versus 7.5% aprotinin; *P *= 0.02). Multivariate logistic regression identified EURO score II and CPB time as additional risk factors for this increased mortality.

**Conclusions:**

The use of high-dose TXA is questioned, as our data suggest an association between higher mortality and minor efficiency while the safety profile of this drug is not consistently improved. Further confirmatory prospective studies evaluating the efficacy and safety profile of TXA are urgently needed to find a safe dosage for this antifibrinolytic drug.

## Introduction

Antifibrinolytic agents are commonly used during cardiac surgery to minimize bleeding and to reduce exposure to blood products. In 2006, the use of aprotinin became controversial when the drug was associated with an increased risk of renal failure, myocardial infarction, stroke, and death in a large observational study [1]. Retrospective analyzed data from the Mc SPI database published by Mangano *et al. *[1,2] seemed to show that the use of aprotinin was associated with the increased risk of postoperative complications after cardiac surgery and even with an increased mortality. The authors of this study concluded that the association between aprotinin and serious end-organ damage indicates that its continued use is not prudent [1]. In contrast, the less-expensive generic medications ε-aminocaproic acid and tranexamic acid (TXA) would be safe alternatives. However, this conclusion might be problematic, being drawn for all types of cardiac surgical patients from a retrospective study. However, subsequent published cohort studies also linked aprotinin to an increased risk of morbidity and mortality [2-5].

In 2007, data from the BART trial were published [6]. The BART trial originally was designed as a multicenter trial looking into whether aprotinin was superior to TXA and aminocaproic acid in decreasing the risk of massive postoperative bleeding in patients undergoing high-risk cardiac surgery. The trial was terminated early because of a higher rate of death in patients receiving aprotinin [6]. Since aprotinin has been withdrawn from the market in many countries, TXA has become the routine antifibrinolytic therapy of choice. Recently, however, evidence indicated that the application of TXA might be associated with morbidity as well. Noteworthy are especially neurologic complications that have been shown by recent studies, especially in pediatric patients and in patients undergoing open-heart procedures [7-9]. From this point of view, it is crucial to know the safety profile of different antifibrinolytic therapies in cardiac surgery to prevent any harm in patients at risk. This is especially important in the context of the work of Karkouti [10], showing that from their single-center experience, high-risk patients given TXA had an excessive complication rate.

Therefore, the aim of this prospective observatory study was to evaluate the safety and efficiency profile of TXA compared with aprotinin in patients undergoing cardiac surgery with CPB and in patients with open-heart procedures, as was suggested recently [7].

## Materials and methods

### Group assignment

**Figure 1:**
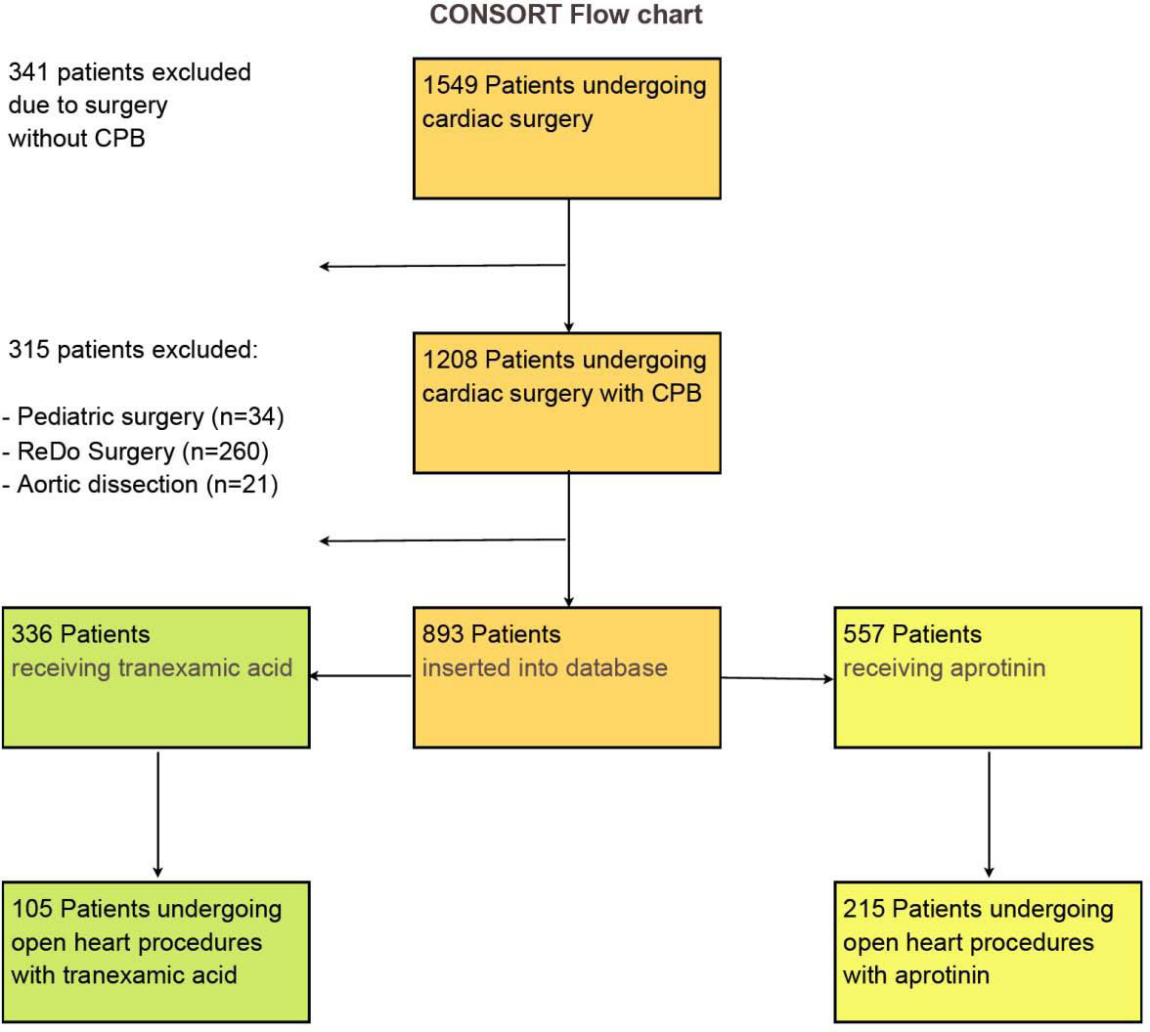
**CONSORT Flow chart of the study design**.

After publication of the first Mangano article raising concerns about the safety profile of aprotinin, we changed our routine administration of antifibrinolytics. On July 1, 2006, we discontinued the use of aprotinin. From that time, we prospectively collected anonymized data in a database evaluating parameters of efficiency and safety. Data of 336 patients receiving TXA were compared with retrospectively collected data from 557 consecutive patients receiving aprotinin undergoing cardiac surgery with cardiopulmonary bypass (CPB) during the 6 months before the change in our antifibrinolytic practice. Patients gave consent for observational studies in our institution. The local ethics committee approved this observational study. A subgroup of 320 patients undergoing open-heart procedures (105 receiving TXA and 215 receiving aprotinin) was analyzed separately (Figure 1).

### Anesthetic, cardiopulmonary bypass, and intensive care management

Our standard anesthetic practice for patients undergoing cardiac surgery with CPB is to use etomidate, sufentanil, and pancuronium for induction and sevoflurane, with propofol and sufentanil infusion for maintenance. In all patients, a radial artery was punctured before induction. The radial artery catheter was used for measurement of arterial blood pressure and to obtain blood samples for point-of-care blood gas analysis (ABL-700 series; Radiometer, Copenhagen, Denmark). A central venous catheter was inserted via the right internal jugular vein.

The prime for the cardiopulmonary bypass circuit consisted of 600 ml of crystalloid fluid, 500 ml of 6% hydroxyethylstarch (HES) solution (Voluven; Fresenius-Kabi, Bad Homburg, Germany). A total dose of 50,000 KIU aprotinin per kilogram bodyweight was administered during CPB in the aprotinin group. The TXA group received 50 mg/kg bodyweight as a bolus before CPB and 50 mg/kg bodyweight into the CPB circuit. Pump flow was adjusted to maintain a mean arterial pressure (MAP) of 55 to 60 mm Hg and a venous oxygen saturation >75% during CPB. When the MAP could not be maintained by adjusting the pump flow, norepinephrine was used. During cardiopulmonary bypass, a partial arterial pressure of oxygen (paO_2_) of 150 to 250 mm Hg was maintained. Body temperature was kept between 35.5 and 36.0°C during CPB, and intermittent antegrade warm-blood cardioplegia was used as described by Calafiore [11]. After surgery, all patients were transferred to the ICU. ICU management and transfusion practice did not differ between patients of both groups. After ICU treatment, all patients were first transferred to the intermediate care unit (IMCU).

### Database management

The prospective data collection begun on July 1, 2006, when the first patient received routinely TXA for cardiac surgery, according to our revised standard operating procedures. Into the same database, we retrospectively collected consecutive data from the last 6 months from patients receiving aprotinin for cardiac surgery with CPB (beginning from January 2 until June 30, 2006).

Safety was evaluated by routinely monitored myocardial biomarkers (creatinine kinase (CK) and isoenzyme MB (CK-MB), creatinine, and the diagnosis of myocardial infarction, ischemic stroke, intracerebral hemorrhage, convulsive seizures, and acute renal failure during ICU and IMCU stay. Efficiency was documented in this database by the need for transfusion of blood products (erythrocyte concentrates, fresh frozen plasma, and platelet concentrates) and total postoperative blood loss (first 6 h after surgery, 24 h after surgery, until 48 hours after surgery), as well as the number of surgical reexplorations for bleeding. We documented in-hospital mortality, duration of ventilation, ICU treatment, and hospital stay as further outcome parameters. All complications were graded as early (during ICU stay) and late (during further hospital stay). Transfusion was guided by our written and published local standard operating procedures.

The diagnosis of myocardial infarction was based on the presence of new Q waves in two contiguous electrocardiogram leads and an increase of myocardial creatine kinase (CK-MB) above 10% of total creatine kinase (CK) or confirmed graft occlusion within the first 30 days after surgery. Ischemic stroke was defined as a focal neurologic deficit lasting more than 24 h and had to be confirmed by a cerebral CT scan and the attending neurologic consultant. Neurologic disability was defined as any newly developed neurologic impairment that lasted longer than 24 h and had to be confirmed by a neurologic consultant. Convulsive seizures were defined as clinically apparent seizures. All patients with seizures underwent routine cerebral CT scan to exclude ischemia or bleeding. Acute renal failure was defined as a decrease in urine output below 500 ml/24 h, the need for at least one dialysis treatment, a doubling of the baseline serum creatinine level, or a postoperative serum creatinine level of more than 150 μmol/L (1.7 mg/dl) with normal creatinine before surgery. Thrombembolic cause of death was defined as death due to a thromboembolic event (for example, myocardial infarction, ischemic stroke, pulmonary embolism).

The group of patients undergoing open-heart procedures was defined as valve surgery, CABG with atrial ablation procedures on the ascending aorta, ventriculotomy, and atrial and ventricular septal defect repair.

### Statistical methods

Results were expressed as mean ± standard deviation (SD) in case of continuous variables. Absolute and relative frequencies were used for categoric and dichotomous variables. The effect on outcome variables was analyzed by using the Exact χ^2 ^test for categoric and dichotomous variables. A check for normal distribution did not reveal substantial deviations from normality (Lilliefors test); therefore, we applied the *t *test for comparisons of independent groups in case of continuous variables.

Multivariate backward stepwise logistic regression analysis with mortality as the response was accomplished to investigate the impact of interesting clinical characteristics such as age, CPB time, Euro II Score, type of surgery, creatinine, hemoglobin, and type of antifibrinolytic. Odds ratios (ORs) with 95% confidence intervals (CIs) and the corresponding *P *values were determined. Changes in blood loss over time were analyzed by using nonparametric analysis of longitudinal data in a two-factorial design (first factor: TXA vs. aprotinin; second factor: Time). Therefore, we compared all the time points simultaneously on the corresponding response curves. The *P *values for differences between groups (first factor) were marked with *P*_groups_, for changes in time (second factor) with *P*_time_, and for interactions (differences increase with time) with *P*_intact_.

As this study was designed as an exploratory investigation, no statistical sample size (power) calculation was conducted.

A *P *value < 0.05 (two-sided) was considered statistically significant. Multiple testing for differences between the groups in question was regarded as exploratory and not confirmatory; therefore, no adjustments for multiplicity were made. Confirmatory studies should use data from this study for the design of an adequately powered trial confirming our results.

Statistical analysis was carried out by using the Software Package for Social Sciences, 16.0 SPSS^® ^for Macintosh (SPSS, Inc., Chicago, IL).

## Results

During the 12-month study period, we included 893 patients undergoing cardiac surgery with CPB into our database, with a group of 557 consecutive patients receiving aprotinin and 336 patients receiving TXA (Figure 1).

Patient's baseline characteristics are shown in Table 1. No significant differences were found with regard to baseline characteristics, with the exception of preoperative hemoglobin being significantly lower in patients in the TXA group (13.1 mg/dL ± 2.0 versus 13.6 mg/dL ± 1.8; *P *< 0.01). In Table 2, types of surgery are displayed. Surgery-type related data (Table 2) did not differ between groups (*P *= 0.15). Also the EUROSCORE II did not differ (6.3 ± 3.9 aprotinin group versus 5.8 ± 3.7 TXA group; *P *= 0.08). Furthermore, no significant differences were noted between both groups with regard to comorbidities as diabetes mellitus (*P *= 0.71), peripheral vascular disease (*P *= 0.76), renal insufficiency (*P *= 0.10), and COPD (*P *= 0.47). No significant difference existed between both groups concerning the treatment with and, if treated, how many days before surgery the vitamin K antagonist, clopidogrel, and acetylsalicylic acid were paused.

**Table 1:** Baseline characteristics of the patients in the aprotinin and tranexamic acid groups

	Aprotinin		Tranexamic acid		
			
	Mean	SD	Mean	SD	*P*
**Age (years)**					
All patients	68	11	67	11	0.09
Open-heart procedures	69	11	68	13	0.25
**Height (cm)**					
All patients	171	9	172	9	0.88
Open-heart procedures	171	9	170	11	0.71
**Weight (kg)**					
All patients	80	15	80	16	0.74
Open-heart procedures	77	17	77	15	0.94
**Ejection fraction preop (%)**					
All patients	55	15	53	15	0.20
Open-heart procedures	53	14	51	16	0.18
**Creatinine preop (mg/dL)**					
All patients	1.18	0.76	1.24	0.87	0.28
Open-heart procedures	**1.14**	0.46	**1.31**	1.03	**0.04**
**Platelets preop (/nL)**					
All patients	235	88	239	80	0.51
Open-heart procedures	241	86	241	88	0.97
**WBC preop (/nL)**					
All patients	8.3	4.2	8.1	2.9	0.37
Open-heart procedures	8.4	4.2	8.1	3.5	0.55
**Hemoglobin preop (mg/dL)**					
All patients	**13.6**	1.8	**13.1**	2.0	**< 0.01**
Open-heart procedures	**13.2**	1.9	**12.5**	1.8	**< 0.01**
**Prothrombin time preop (%)**					
All patients	93	15	93	14	0.69
Open-heart procedures	91	17	88	16	0.15
**PTT preop (s)**					
All patients	41.1	23.7	42.3	24.0	0.49
Open-heart procedures	40.6	22.2	41.3	17.8	0.76
**AT III preop (%)**					
All patients	97	15	95	17	0.17
Open-heart procedures	97	14	93	19	0.05

AT III, antithrombin; PTT, partial thromboplastin time; SD, standard deviation; WBC, white blood cell count.

**Table 2:** Surgical, ICU, and outcome data of patients receiving aprotinin and tranexamic acid

		Aprotinin		Tranexamic acid		
				
CABG	*n*	349		231		
	% in group	63.1%		69.0%		
Valve	*n*	106		48		
	% in group	19.2%		14.3%		
Double valve	*n*	11		2		
	% in group	2.0%		0.6%		
CABG plus valve	*n*	68		41		
	% in group	12.3%		12.2%		
Other	*n*	19		13		
	% in group	3.4%		3.9%		
				
		Mean	SD	Mean	SD	***P***
**Duration of surgery (min)**						
All patients		206	60	211	71	0.28
Open-heart procedures		**210**	65	**235**	79	**< 0.01**
**CPB time (min)**						
All patients		89	42	88	46	0.79
Open-heart procedures		104	47	114	53	0.07
**Cross-clamp time (min)**						
All patients		59	34	57	36	0.37
Open-heart procedures		77	40	85	43	0.08
**Euroscore II**						
All patients		6.3	3.9	5.8	3.7	0.08
Open-heart procedures		7.7	3.6	7.3	3.7	0.42
**ICU treatment (days)**						
All patients		3.1	9.5	3.5	8.1	0.51
Open-heart procedures		4.3	13.8	5.7	11.8	0.38
**Hospital stay (days)**						
All patients		17.0	17.1	18.9	18.6	0.11
Open-heart procedures		20.8	20.8	23.6	25.1	0.28
**Mechanical ventilation (h)**						
All patients		25.6	128.0	45.4	187.3	0.06
Open-heart procedures		36.3	188.4	83.0	263.6	0.07
**APACHE II (admission ICU)**						
All patients		19.4	6.8	19.3	7.0	0.89
Open-heart procedures		20.2	7.3	19.8	6.7	0.60
**SAPS II (admission ICU)**						
All patients		**34.3**	12.4	**36.4**	12.5	**0.02**
Open-heart procedures		36.7	13.0	39.7	13.1	0.06

APACHE II, acute physiology and chronic health evaluation score II; CABG, coronary artery bypass graft; CPB, cardiopulmonary bypass; ICU, intensive care unit; SAPS, simplified acute physiological score; SD, standard deviation.

Analysis of biochemical safety data revealed no differences between both groups, with the exception of a slight increase of creatinine in patients receiving TXA immediately after surgery (1.2 mg/dL ± 0.8 versus 1.1 mg/dL ± 0.7; *P *= 0.02). The PT ratio and PTT were slightly different between both groups (Table 3). Acute renal failure was identical between groups (9.4% aprotinin versus 11.6% TXA, *P *= 0.31). However, acute renal failure was seen more often in patients receiving TXA (13.7%) compared with patients receiving aprotinin (8.5%; *P *= 0.02).

**Table 3:** Biochemical data of patients receiving aprotinin and tranexamic acid

	Aprotinin		Tranexamic acid		
			
	Mean	SD	Mean	SD	*P*
**Creatinine after surgery (mg/dL)**					
All patients	**1.1**	0.7	**1.2**	0.8	**0.02**
Open-heart procedures	**1.08**	0.50	**1.30**	0.73	**< 0.01**
**Creatinine POD 1 (mg/dL)**					
All patients	1.5	5.3	2.2	14.0	0.29
Open-heart procedures	1.37	1.83	1.42	0.65	0.77
**CK after surgery (U/mL)**					
All patients	521	635	474	782	0.33
Open-heart procedures	521	605	589	1307	0.53
**CK-MB after surgery (U/mL)**					
All patients	59	64	54	69	0.27
Open-heart procedures	59	64	77	109	0.39
**CK POD 1 (U/mL)**					
All patients	1013	1496	878	1131	0.16
Open-heart procedures	1032	1462	914	1224	0.48
**CK-MB POD 1 (U/mL)**					
All patients	55	69	52	80	0.51
Open-heart procedures	59	64	67	103	0.42
**WBC after surgery (/nL)**					
All patients	12.6	5.5	12.5	5.4	0.80
Open-heart procedures	13.6	5.7	13.8	6.3	0.74
**WBC POD 1 (/nL)**					
All patients	14.0	4.4	13.8	6.9	0.63
Open-heart procedures	**14.4**	4.4	**13.4**	4.2	**0.04**
**Hemoglobin after surgery (g/dL)**					
All patients	10.2	1.2	10.5	7.3	0.27
Open-heart procedures	10.1	1.4	11.1	12.9	0.26
**Hemoglobin POD 1 (g/dL)**					
All patients	10.4	1.2	10.3	3.8	0.77
Open-heart procedures	10.3	1.1	10.7	6.6	0.44
**Platelets after surgery (/nL)**					
All patients	144	50	153	56	0.01
Open-heart procedures	143	55	149	62	0.35
**Platelets POD 1 (/nL)**					
All patients	158	55	160	57	0.53
Open-heart procedures	147	59	145	59	0.75
**PT ratio after surgery (%)**					
All patients	**65**	10	**64**	10	**0.03**
Open-heart procedures	**64**	11	**61**	10	**< 0.01**
**PT ratio POD 1 (%)**					
All patients	**75**	12	**74**	11	**0.04**
Open-heart procedures	73	13	71	13	0.12
**aPTT after surgery (s)**					
All patients	**51.0**	16.7	**41.0**	14.5	**< 0.01**
Open-heart procedures	**53.6**	19.0	**43.4**	21.0	**< 0.01**
**aPTT POD 1 (s)**					
All patients	**44.0**	15.1	**41.2**	10.3	**< 0.01**
Open-heart procedures	46.6	17.9	42.7	12.2	0.05
**AT III after surgery (%)**					
All patients	67	12	68	13	0.37
Open-heart procedures	69	12	69	15	0.67
**AT III POD 1 (%)**					
All patients	76	14	74	14	0.31
Open-heart procedures	78	13	75	15	0.34

AT III, antithrombin; CK, creatinine kinase; CK-MB, creatinine kinase isoenzyme MB; POD, postoperative day; PT, prothrombin ratio; PTT, partial thromboplastin time; SD, standard deviation; WBC, white blood cell count.

Patients receiving aprotinin had a higher rate of late events of ischemic stroke (3.4% versus 0.9%; *P *= 0.02) and late neurologic disability (5.8% versus 2.4%; *P *= 0.02). The rate of postoperative convulsive seizures in the ICU was increased in tendency in patients receiving TXA (2.7% versus 0.9%; *P *= 0.05) compared with patients receiving aprotinin. No difference regarding myocardial infarction, intracerebral hemorrhage, and acute renal failure was observed (Table 4). In-hospital mortality in all patients did not differ between both groups (6.9% aprotinin versus 8.7% TXA; *P *= 0.34).

**Table 4:** Safety data of patients receiving aprotinin and tranexamic acid

			Aprotinin	Tranexamic acid	*P*
Myocardial infarction (ICU)	All patients	*N*	8	6	0.78
	Open-heart procedures		**0**	**2**	**0.04**
	All patients	% in group	1.4%	1.8%	
	Open-heart procedures		**0.0%**	**1.9%**	

Myocardial infarction (late)	All patients	*N*	7	5	0.77
	Open-heart procedures		1	1	0.60
	All patients	% in group	1.3%	1.5%	
	Open-heart procedures		0.5%	1.0%	

Seizures (ICU)	All patients	*N*	5	9	0.05
	Open-heart procedures		**4**	**7**	**0.04**
	All patients	% in group	0.9%	2.7%	
	Open-heart procedures		**1.9%**	**6.7%**	

Seizures (late)	All patients	*N*	6	1	0.20
	Open-heart procedures		3	1	0.74
	All patients	% in group	1.1%	0.3%	
	Open-heart procedures		1.4%	1.0%	

Ischemic stroke (ICU)	All patients	*N*	21	9	0.45
	Open-heart procedures		10	6	0.68
	All patients	% in group	3.8%	2.7%	
	Open-heart procedures		4.7%	5.7%	

Ischemic stroke (late)	All patients	*N*	**19**	**3**	0.02
	Open-heart procedures		9	1	0.12
	All patients	% in group	**3.4%**	**0.9%**	
	Open-heart procedures		4.2%	1.0%	

Neurologic disability (ICU)	All patients	*N*	21	11	0.85
	Open-heart procedures		12	9	0.34
	All patients	% in group	3.8%	3.3%	
	Open-heart procedures		5.6%	8.6%	

Neurologic disability (late)	All patients	*N*	**32**	**8**	**0.02**
	Open-heart procedures		**15**	**1**	**0.03**
	All patients	% in group	**5.8%**	**2.4%**	
	Open-heart procedures		**7.0%**	**1.0%**	

Intracerebral hemorrhage (ICU)	All patients	*N*	3	1	1.00
	Open-heart procedures		2	1	1.00
	All patients	% in group	0.5%	0.3%	
	Open-heart procedures		0.9%	1.0%	

Intracerebral hemorrhage (late)	All patients	*N*	1	0	1.00
	Open-heart procedures		0	0	n/a
	All patients	% in group	0.2%	0.0%	
	Open-heart procedures		0.0%	0.0%	

Mortality (in-hospital)	All patients	*N*	38	29	0.34
	Open-heart procedures		**16**	**17**	**0.02**
	All patients	% in group	6.9%	8.7%	
	Open-heart procedures		**7.5%**	**16.2%**	

Patients in the TXA group showed a trend for prolonged need of mechanical ventilation (45.4 h ± 187.3 versus 25.6 h ± 128.0; *P *= 0.06. This led in tendency to a prolonged hospital stay of 2 days compared with the aprotinin group (18.9 days ± 18.6 versus 17.0 days ± 17.1; *P *= 0.11) (Table 2).

**Figure 2:**
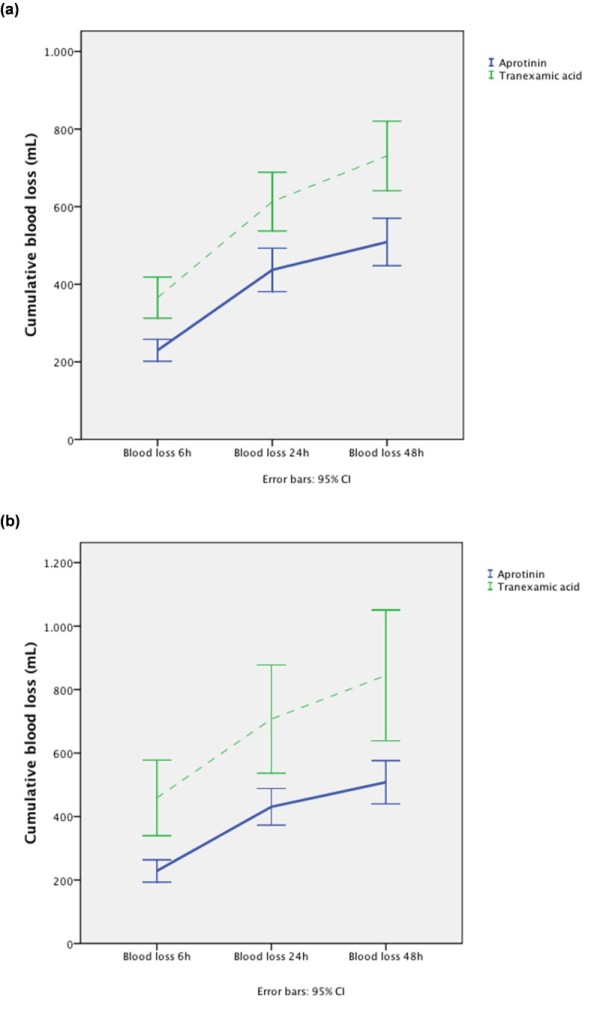
**Cumulative blood loss for the first 48 h after surgery for (a) all patients and (b) patients undergoing open-chamber procedures**. **(a) **All patients (*P*_groups _< 0.01; *P*_time _< 0.01; *P*_intact _< 0.01). **(b) **Patients with open-heart procedures (*P*_groups _< 0.01; *P*_time _< 0.01; *P*_intact _< 0.01).

**Figure 3:**
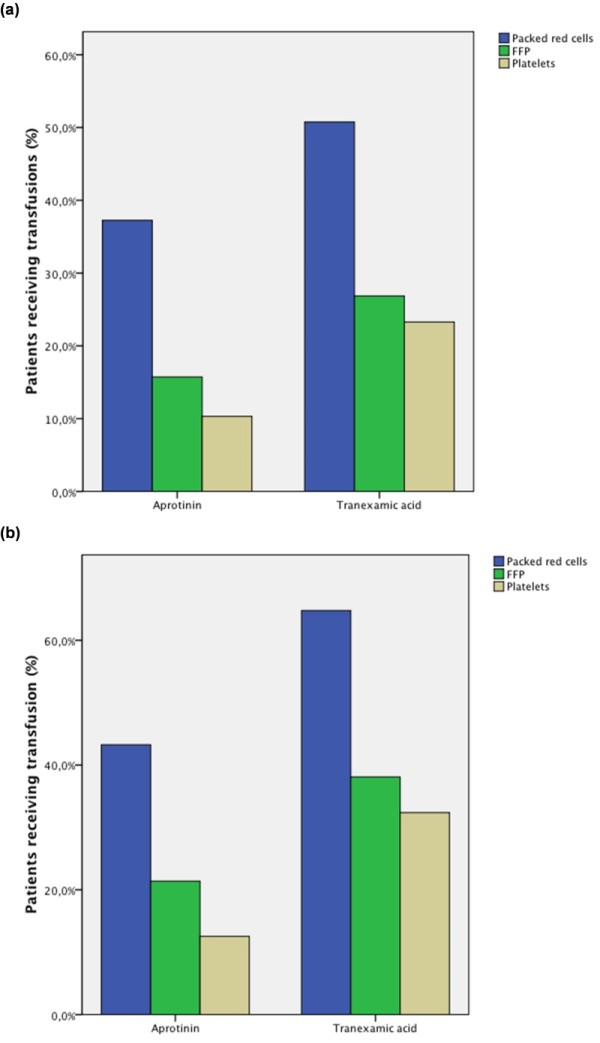
**Percentage of patients receiving transfusions for all patients and for patients undergoing open chamber procedures**. **(a) **All patients (*P*_groups _< 0.01; *P*_time _< 0.01; *P*_intact _< 0.01). **(b) **Patients with open-heart procedures (*P*_groups _< 0.01; *P*_time _< 0.01; *P*_intact _< 0.01).

Patients being treated with TXA had increased cumulative drainage losses at 6, 24, and 48 h after surgery (Figure 2a; Table 5) compared with patients receiving aprotinin (*P*_groups _< 0.01; *P*_time _< 0.01; *P*_intact _< 0.01). These patients did receive significantly more packed red cells, units of fresh frozen plasma, and platelet concentrates (Table 5) compared with patients receiving aprotinin. The use of aprotinin was associated with a decreased risk of being transfused with packed red cells (*P *< 0.01), units of fresh frozen plasma (*P *< 0.01), and platelet concentrates (*P *< 0.01) (Figure 3a). Furthermore, the use of TXA was associated with an increased rate of repeated thoracotomy for bleeding (6.9% versus 2.4%; *P *< 0.01).

**Table 5:** Blood loss, transfusion, and coagulation-related data for patients receiving aprotinin and tranexamic acid

		Aprotinin		Tranexamic acid		
				
		Mean	SD	Mean	SD	*P*
**Blood loss first 6 h (mL)**						
All patients		**230**	338	**366**	492	**< 0.01**
Open-heart procedures		**229**	260	**459**	616	**< 0.01**
**Blood loss first 24 h (mL)**						
All patients		**437**	669	**613**	705	**< 0.01**
Open-heart procedures		**431**	431	**707**	881	**< 0.01**
**Blood loss 48 h (mL)**						
All patients		**72**	159	**118**	223	**< 0.01**
Open-heart procedures		**77**	177	**137**	284	**0.02**
**Packed red blood surgery (units)**						
All patients		**0.5**	1.2	**0.7**	1.5	**0.02**
Open-heart procedures		0.6	1.2	0.8	1.4	0.19
**Packed red blood first 24 h (units)**						
All patients		**0.7**	2.1	**1.3**	2.4	**< 0.01**
Open-heart procedures		**0.8**	1.8	**1.9**	2.9	**< 0.01**
**FFP surgery (units)**						
All patients		**0.3**	1.1	**0.4**	1.4	**0.09**
Open-heart procedures		**0.5**	1.5	**0.8**	1.6	**0.13**
**FFP first 24 h (units)**						
All patients		**0.8**	4.4	**1.6**	5.0	**< 0.01**
Open-heart procedures		**1.0**	3.2	**2.8**	7.3	**< 0.01**
**Platelet concentrates surgery (units)**						
All patients		**0.1**	0.5	**0.2**	0.6	**< 0.01**
Open-heart procedures		**0.1**	0.5	**0.3**	0.7	**< 0.01**
**Platelet concentrates first 24 h (units)**						
All patients		**0.2**	0.8	**0.4**	1.2	**< 0.01**
Open-heart procedures		**0.2**	0.9	**0.6**	1.7	**< 0.01**
**PPSB (IU)**						
All patients		14.1	154.7	30.4	186.1	0.16
Open-heart procedures		19.5	168.0	57.1	269.9	0.13
**AT III (IU)**						
All patients		23.5	200.5	41.8	275.8	0.26
Open-heart procedures		30.2	237.3	38.1	237.1	0.78
**F XIII (IU)**						
All patients		9.0	130.0	14.2	161.8	0.60
Open-heart procedures		11.6	120.3	23.8	244.0	0.55
**aFVII (IU)**						
All patients		3.1	46.8	5.0	39.1	0.52
Open-heart procedures		3.4	36.8	13.7	65.1	0.07
**Desmopressin (μg/kg)**						
All patients		0.3	2.4	0.2	1.9	0.58
Open-heart procedures		0.5	3.2	0.4	2.5	0.71
**Rethoracotomy (bleeding)**						
All patients	*N*	**13**		**23**		**< 0.01**
Open-heart procedures		**7**		**15**		**< 0.01**
All patients	% in group	**2.4%**		**6.9%**		
Open-heart procedures		**3.3%**		**14.3%**		

aFVII, activated factor VII concentrate; AT III, antithrombin; FFP, fresh frozen plasma; F XIII, factor XIII concentrate; ICU, intensive care unit; PPSB, coagulation factor concentrate (prothrombin, factor VII, factor X, factor IX); OR, odds ratio; SD, standard deviation.

### Subgroup with open-heart procedures

In the subgroup of patients undergoing open-heart procedures, 320 patients (105 receiving TXA and 215 receiving aprotinin) were analyzed. In this group, patients receiving TXA had significantly higher preoperative creatinine (1.31 ± 1.03 mg/dL versus 1.14 ± 0.46 mg/dL; *P *= 0.04) and again significantly lower levels of hemoglobin (12.5 ± 1.8 mg/dL versus 13.2 ± 1.9 mg/dL; *P *> 0.01).

Patients with open-heart procedures receiving TXA had increased duration of surgery (235 min ± 79 versus 210 min ± 65; *P *> 0.01); however, no difference between duration of CPB and aortic cross-clamping time and no difference between EURO score, APACHE II, and SAPS score on admission to the ICU was detectable (Table 2). The type of surgery, type and duration of treatment with vitamin K antagonists, clopidogrel and aspirin, as well as other comorbidities did not differ between both groups.

Patients with open-heart procedures in the TXA group showed a trend for prolonged need of mechanical ventilation (83.0 h ± 263.6 versus 36.3 h ± 188.4; *P *= 0.07. However, in this subgroup, no significant difference regarding duration of ICU treatment and hospital stay was detectable (Table 2).

Analysis of biochemical safety data is shown in Table 3. In this subgroup, an increase of creatinine in patients receiving TXA immediately after surgery was seen (1.30 mg/dL ± 0.73 versus 1.08 mg/dL ± 0.50; *P *< 0.01). The WBC, PT ratio, and aPTT were slightly different between both groups (Table 3). Acute renal failure was identical between groups (9.8% aprotinin versus 13.3% TXA; *P *= 0.35). However, acute renal failure was seen more often in patients receiving TXA (20.0%) compared with patients receiving aprotinin (11.2%; *P *= 0.04).

Even if patients with open-heart procedures receiving aprotinin did not show a significant higher rate of events of ischemic stroke (4.2% versus 1.0%; *P *= 0.12), we detected a higher rate of late neurologic disability (7.0% versus 1.0%; *P *= 0.03). The rate of postoperative convulsive seizures was increased in patients receiving TXA (6.7% versus 1.9%; *P *= 0.04) compared with patients treated with aprotinin. No difference regarding intracerebral hemorrhage and acute renal failure was observed. A slight increase in the rate of myocardial infarction was seen in patients receiving TXA (Table 4).

Notably, in patients with open-heart procedures, in-hospital mortality was more than twofold increased in patients receiving TXA (16.2% TXA versus 7.5% aprotinin; *P *= 0.02). The leading cause of death was thromboembolic events (21 of 38 deaths) in patients receiving TXA compared with the aprotinin group (11 of 29 deaths). In the multivariate backward stepwise logistic regression, aprotinin as antifibrinolytic, higher EURO score II, and prolonged CPB time were identified as independent risk factors for the excess mortality in the open-heart procedures group (Table 6).

**Table 6:** Multivariate backward stepwise logistic regression analysis of the increased mortality in open-heart procedures

		B	SE	**Sig**.	Odds ratio (OR)	95.0% CI for OR	
						
						Lower	Upper
Step 1	Age	-0.043	0.018	0.019	0.958	0.924	0.993
	CPB time	0.012	0.004	0.002	1.012	1.004	1.020
	EURO Score II	0.278	0.063	0.000	1.321	1.168	1.493
	Creatinine preop	0.146	0.207	0.481	1.157	0.772	1.734
	Hemoglobin preop	-0.148	0.125	0.238	0.863	0.675	1.103
	Antifibrinolytic	-0.788	0.437	0.071	0.455	0.193	1.070
	Constant	-0.940	1.959	0.631	0.391		

Step 2	Age	-0.041	0.018	0.023	0.959	0.926	0.994
	CPB time	0.012	0.004	0.001	1.012	1.005	1.020
	EURO Score II	0.276	0.062	0.000	1.318	1.167	1.489
	Hemoglobin preop	-0.175	0.119	0.142	0.840	0.665	1.060
	Antifibrinolytic	-0.832	0.431	0.054	0.435	0.187	1.013
	Constant	-0.534	1.866	0.775	0.586		

**Step 3**	**Age**	**-0.044**	**0.018**	**0.014**	**0.957**	**0.923**	**0.991**
	**CPB Time**	**0.011**	**0.004**	**0.003**	**1.011**	**1.004**	**1.019**
	**EURO Score II**	**0.300**	**0.060**	**0.000**	**1.350**	**1.200**	**1.518**
	**Antifibrinolytic**	**-0.947**	**0.424**	**0.025**	**0.388**	**0.169**	**0.890**
	**Constant**	**-2.554**	**1.302**	**0.050**	**0.078**		

CI, confidence interval; CPB, cardiopulmonary bypass; OR, odds ratio; SD, standard deviation; SEM, standard error of the mean.

With regard to the efficacy of antifibrinolytic therapy, open-heart procedures being treated with TXA showed increased cumulative drainage losses at 6, 24, and 48 h after surgery (Figure 2b; Table 5) compared with patients receiving aprotinin (*P*_groups _< 0.01; *P*_time _< 0.01; *P*_intact _< 0.01) and did receive significantly more packed red blood cells (PRBCs), units of fresh frozen plasma (FFP), and platelet concentrates (PCs) (Figure 3b). Again, the aprotinin patients had a decreased risk of being transfused with PRBCs (*P *< 0.01), units of FFP (*P *< 0.01), and PC (*P *< 0.01) (Figure 3b; Table 5). The need for repeated thoracotomy for bleeding in patients receiving TXA was almost 5 times higher (14.3% versus 3.3%; *P *< 0.01) compared with aprotinin-treated patients (Table 5).

## Discussion

The two major findings of our study are as follows: first, in the overall cardiac surgery population studied, the administration of high-dose TXA showed a strong trend toward an association with convulsive seizures, whereas aprotinin was associated with a higher rate of stroke and neurologic disability after cardiac surgery with CPB. Second, in patients undergoing open-heart cardiac surgery treated with TXA, an increased mortality and a significant increase in convulsive seizures compared with patients receiving aprotinin was observed.

At our institution, aprotinin has been used for many years as the primary antifibrinolytic in patients undergoing cardiac surgery with CPB. Several studies and meta-analyses showed its superiority compared with other antifibrinolytic drugs, especially in high-risk patients undergoing cardiac surgery [8,12-14]. However, is has been criticized that the safety profile of aprotinin was not thoroughly investigated [1-3]. Early reports linked its use with a higher incidence of graft occlusion after CABG surgery and renal failure [15,16]. Recently the use of aprotinin was associated with a significantly increased risk for renal failure, myocardial ischemia, stroke [1], and an impaired 5-year mortality [2]. In comparison, the same studies suggested TXA to be the safer choice of antifibrinolytic treatment. So far, only a few studies and case reports have reported the safety profile of TXA [3,17].

Our results support an association between convulsive seizures and the use of TXA, and especially patients undergoing open-heart procedures seem to be at risk. This supports recent data from the literature indicating an increased rate of seizures in patients receiving TXA for open-heart surgery (7.9% compared with 1.2% (*P *< 0.01) compared with aprotinin-treated patients) [7]. Earlier case reports and experimental data indicated that TXA is linked to an epileptogenic effect if it is applied to the central nervous system [18-20]. It was hypothesized that this effect might occur in part because of binding of TXA to the γ-aminobutyric acid (GABA) binding site of GABA-(A) receptors, as shown in membranes from rat cerebral cortex [21]. A recent report of Murkin *et al. *[9] linked the use of TXA to seizures. In two separate centers, they observed a notable increase in the incidence of postoperative convulsive seizures from 1.3% to 3.8% in patients having undergone major cardiac surgical procedures. These events were temporally coincident with the introduction of high-dose TXA therapy after discontinuation of aprotinin from general clinical use. They concluded that use of high-dose TXA in older patients in conjunction with cardiopulmonary bypass and open-heart cardiac surgery is associated with clinical seizures in susceptible patients [9].

Furthermore, we could show an association between renal failure and treatment with TXA. This could be an effect of the increased blood loss, need for transfusion, and need of repeated thoracotomy. All these factors have been linked to unfavorable outcomes in cardiac surgery patients [22,23]. Conversely, this finding is surprising, as the use of TXA was associated with a better renal outcome in previous studies [1,3].

Our findings that patients receiving aprotinin had a more than threefold higher rate of ischemic stroke and neurologic disability are in line with those of previous studies [1]. One hypothesis for explanation of the impaired neurologic outcome with aprotinin may be the occurrence of microvascular thrombosis, as described by Sundt *et al. *[24], who reported platelet-fibrin thrombi among multiple vessels, including the cerebral arteries, on postmortem examination of patients who had received aprotinin. These results confirm earlier results from observational or recent retrospective studies [1,2], but are in contrast with results that report no difference in the incidence of stroke for aprotinin in cardiac surgery [3,10,25]. This might well be explained by the fact that the rates of ischemic stroke in the TXA group were low. Another German center reported a stroke rate for open procedures of 7.4% [26]. The McSPI dataset suggests that patients having combined CABG/valve replacement had permanent neurologic deficit in about 8% [27]. Therefore, the observed neurologic deficit rate for aprotinin might be about as expected.

However, also for TXA, an association with a thromboembolic risk must be hypothesized, as the leading cause of death in the group of patients treated with TXA was thromboembolic.

Our result that patients treated with TXA had increased cumulative drainage loss compared with patients receiving aprotinin is in accordance with previous studies and meta-analysis [8,12-14,28,29]. It has been shown that aprotinin is superior to TXA in reducing postoperative blood loss. One explanation for this may be its potential to inhibit plasmin, the final enzyme of fibrinolysis [30]. Our results confirm that the total number of transfusions and the risk of being transfused were significantly lower in the aprotinin group, as shown by others [14]. The increased bleeding might be responsible for the longer duration of surgery to achieve surgical hemostasis. Furthermore, the increased blood loss could explain in part the somewhat prolonged ventilation and the trend for prolonged hospital stay in patients receiving TXA.

The increased bleeding seen in our patients receiving TXA explains the significantly higher rate of patients transfused with PRBC, FFP, and PC. This was observed in the general patient population and was even more pronounced in patients with open-heart procedures. More than two thirds of patients undergoing open-heart procedures in the TXA group received allogenic PRBC transfusion. In line with this finding is the significantly increased rate of repeated thoracotomies in the TXA group. As bleeding and reoperation for bleeding has a major impact on outcome, this increased bleeding and need for transfusion, as well as the increased rate for reoperation seen in TXA patients, might in part be also responsible for the increased mortality seen in the TXA open-heart procedures subgroup [23].

The results of our study are in line with recent data [10] indicating that, compared with TXA, the safety profile of aprotinin is better in high-risk cardiac surgery patients. It seems that increased bleeding is associated with a higher risk of complications and mortality after cardiac surgery [23]. Although the effect of aprotinin on mortality is still considered controversial [6,7], an increased mortality might be found in high-risk patients treated with TXA compared with aprotinin-treated patients [10]. These results might indicate a beneficial risk/benefit profile for aprotinin in certain high-risk patients, like those with open-heart procedures analyzed in our study [10].

The strengths of our study include the clinically real-world, unselected nature of the patient population and the prospective and unbiased data collection of patients receiving TXA being treated at a single center. Another strength is that in this study, no crossover between groups requiring some sort of propensity score matching was possible, as aprotinin is no longer available. However, obviously, this study also has all methodologic limitations of a retrospective study with regard to the aprotinin patients. Nonetheless, as we changed our routine practice on July 1, 2006, standard operating procedures in regard to treating cardiac surgical patients were not changed. We had the same surgeons and the same personnel in our operating rooms, ICUs, IMCUs, and normal wards, so that supposedly did not influence our results. As we adhere on a day-by-day basis to written standard operating procedures, all perioperative procedures (anesthetic management, ICU management, transfusion guidelines, and so on) are extremely standardized. Unfortunately, it was not possible to include 260 patients undergoing redo surgery, as all of these patients received aprotinin at that time as part of our standard operating procedures. Therefore, at this time from our own data, we cannot comment on whether aprotinin or tranexamic acid is superior in redo surgery. Although all the deaths were clinically adjudicated in our trial, without detailed investigations like invasive diagnostic or autopsies, a potential source of error remains.

## Conclusions

The association between higher mortality and the minor efficiency of TXA questions the routine administration of high-dose TXA in cardiac surgery. In particular, our finding of the more than twofold increased mortality in patients undergoing open-heart procedures receiving tranexamic acid is worrying. However, our results confirm also that aprotinin is associated with severe neurologic adverse reactions. The safety profile of antifibrinolytic treatment--aprotinin and TXA--warrants further evaluation to answer the question whether the benefit of this treatment outweighs its potential risks. For the future, controlled trials investigating the safety profile of antifibrinolytic therapy are needed. With regard to TXA, the effective and safe dosage as well as the patients who will most likely benefit from this medication must be established.

## Key messages

• The association between higher mortality and the minor efficiency of tranexamic acid questions the routine administration of high-dose tranexamic acid in cardiac surgery. In particular, our finding of the more than twofold increased mortality in patients undergoing open-heart procedures receiving tranexamic acid is worrying.

• Aprotinin and tranexamic acid were associated with neurologic adverse reactions in this retrospective study.

• The safety profile of antifibrinolytic treatment--aprotinin and tranexamic acid--warrants further evaluation to answer the question whether the benefit of this treatment outweighs its potential risks.

## Abbreviations

APACHE II: acute physiology and chronic health evaluation score II; AT III: antithrombin; CABG: coronary artery bypass graft; CI: confidence interval; CK: creatinine kinase; CK-MB: creatinine kinase isoenzyme MB; COPD: chronic obstructive pulmonary disease; CPB: cardiopulmonary bypass; CVP: central venous pressure; EF: ejection fraction; FFP: fresh frozen plasma; FiO_2_: inspiratory oxygen fraction; GABA: γ-aminobutyric acid; Hct: hematocrit; ICU: intensive care unit; IMCU: intermediate care unit; MAP: mean arterial pressure; OR: odds ratio; PC: platelet concentrate; pO_2_: partial pressure of oxygen; POD: postoperative day; PPSB: coagulation factor concentrate (prothrombin, factor VII, factor X, factor IX); PRBCs: packed red blood cells; ROC: receiver operating characteristic; SAPS: simplified acute physiological score; SD: standard deviation; SIRS: systemic inflammatory response syndrome; TXA: tranexamic acid; WBC: white blood cell count.

## Competing interests

The authors declare that they have no competing interests. This work was supported by institutional grants from Charité-Universitätsmedizin, Berlin, Germany.

## Authors' contributions

MS and CvH prepared the manuscript, conceived the study, and performed the statistical analysis. MS, CvH, and VM carried out the data acquisition; KDW prepared the statistical part of the manuscript and performed the statistical analysis. CR and CS drafted the manuscript and helped with the study design and coordination. All authors read and approved the final manuscript.
